# Predictive value of water swallow test score, serum albumin, and systemic immune-inflammation index for stroke-associated pneumonia in patients with acute isolated pontine infarction

**DOI:** 10.3389/fneur.2026.1790221

**Published:** 2026-07-14

**Authors:** Jingjing Ding, Haifeng Jin, Xiaomei Ren, Jieli Geng

**Affiliations:** 1 Department of Neurology, Yizheng People's Hospital, Yangzhou, China; 2 Department of Neurology, Renji Hospital, Shanghai Jiao Tong University School of Medicine, Shanghai, China

**Keywords:** isolated pontine infarction, serum albumin, stroke-associated pneumonia, swallowing function, systemic immune-inflammation index

## Abstract

**Objective:**

To identify independent predictors of stroke-associated pneumonia (SAP) in patients with acute isolated pontine infarction (AIPI) and to investigate the association between SAP and short-term prognosis.

**Methods:**

This retrospective study included 179 consecutive patients with AIPI admitted between March 2021 and March 2024. Baseline clinical data, swallowing function, and serum biomarkers were analyzed. Independent predictors of SAP were determined using multivariate logistic regression, and predictive performance was assessed by receiver operating characteristic (ROC) curve analysis.

**Results:**

SAP occurred in 19 (10.61%) patients. Compared with patients without SAP, those who developed SAP were older and had higher Water Swallow Test (WST) scores, National Institutes of Health Stroke Scale (NIHSS) scores, fibrinogen, C-reactive protein (CRP), neutrophil-to-lymphocyte ratio (NLR), systemic immune-inflammation index (SII), and systemic inflammation response index (SIRI), as well as lower serum albumin levels (all *p* < 0.05). Multivariate logistic regression analysis identified WST score (OR = 2.622, 95% CI: 1.378–4.991, *p* = 0.003), serum albumin level (OR = 0.831, 95% CI: 0.735–0.940, *p* < 0.001), and SII (OR = 1.004, 95% CI: 1.002–1.006, *p* < 0.001) as independent predictors of SAP. The combined model incorporating these three variables demonstrated excellent predictive performance, with an area under the curve (AUC) of 0.938, sensitivity of 89.5%, and specificity of 86.9%. Furthermore, SAP was independently associated with short-term poor functional outcome [modified Rankin Scale (mRS) score ≥ 3] in patients with AIPI (OR = 8.082, 95% CI: 2.922–22.355, *p* < 0.001).

**Conclusion:**

SAP is independently associated with a significantly increased risk of short-term poor functional outcome. WST score, serum albumin, and SII are independent predictors of SAP in patients with AIPI. Their combination provides a simple, cost-effective bedside model for early identification and prevention of post-stroke pneumonia.

## 1 Introduction

Stroke-associated pneumonia (SAP) is a common complication of acute ischemic stroke (AIS), with reported incidence rates ranging from 3.3 to 28.3% (1–3). SAP significantly increases disability, mortality, and healthcare burden (3–5). Among posterior circulation strokes, acute isolated pontine infarction (AIPI) represents a distinctive subtype that carries a relatively high risk of SAP, likely related to frequent dysphagia and impaired airway protection (6–8). However, most previous SAP studies have focused on anterior circulation stroke (2, 9), and predictive indicators specific to AIPI remain insufficiently defined.

Previous studies have identified several risk factors for SAP, including sex, age, the National Institutes of Health Stroke Scale (NIHSS) score, dysphagia, and atrial fibrillation (5, 9–12). Although predictive models such as A2DS2, ISAN, and AIS-APS are available (13, 14), they are primarily based on clinical characteristics and show limited sensitivity for early identification of high-risk patients.

In recent years, increasing attention has been paid to post-stroke immune-inflammatory imbalance. Brain injury can activate the hypothalamic–pituitary–adrenal axis and the sympathetic nervous system, resulting in cytokine release and lymphocyte apoptosis, which lead to acquired immunodeficiency and increased susceptibility to infection (15, 16). Accordingly, inflammatory markers such as the neutrophil-to-lymphocyte ratio (NLR), systemic immune-inflammation index (SII), and systemic inflammation response index (SIRI) have shown independent predictive value for SAP in the general stroke population (17, 18). However, their predictive value in AIPI-specific populations has not been validated. Moreover, nutritional and immune markers, particularly serum albumin, have been closely linked to SAP risk, and hypoalbuminemia has been recognized as an independent predictor (19).

To our knowledge, this is the first study to integrate clinical, functional, and inflammatory parameters to identify predictors of SAP in patients with AIPI. The findings aim to support early risk stratification and guide preventive strategies in this high-risk population.

## 2 Materials and methods

### 2.1 Study population

This retrospective observational cohort study was conducted in the Department of Neurology at Yizheng People’s Hospital. Clinical and demographic data were collected from patients diagnosed with AIPI who were admitted between March 2021 and March 2024. The study protocol was reviewed and approved by the Ethics Committee of Yizheng People’s Hospital. The requirement for informed consent was waived by the Ethics Committee owing to the retrospective nature of the study.

Patients were eligible for inclusion if they met the following criteria: (1) age ≥ 18 years; (2) diagnosis of AIPI confirmed by diffusion-weighted imaging (DWI), showing an acute pontine infarction without extension beyond the pons (20); (3) first-ever ischemic stroke episode; (4) baseline laboratory tests obtained within 24 h of admission and before the diagnosis of SAP; (5) definitive diagnosis of SAP or non-SAP status within 7 days after stroke onset. Exclusion criteria were as follows: (1) active infection or fever within two weeks before admission; (2) antibiotic treatment within one week before hospitalization; (3) severe comorbid organ dysfunction, including renal, hepatic, or cardiac failure, malignancy, or ongoing immunosuppressive therapy; and (4) receipt of intravenous thrombolysis or endovascular intervention.

### 2.2 Clinical data collection

Baseline data included demographic and clinical characteristics, such as age, sex, smoking history, and alcohol use, as well as medical history variables (hypertension, diabetes mellitus, coronary artery disease, atrial fibrillation, transient ischemic attack, or prior stroke). Laboratory parameters included complete blood count, C-reactive protein (CRP), glycated hemoglobin (HbA1c), homocysteine, hepatic and renal function tests, blood glucose, and lipid profile.

Short-term functional prognosis was evaluated according to the Modified Rankin Scale (mRS) scores at 3 months after infarction, and follow-up mRS data were available for all included patients. Patients were predefined as having a favorable functional outcome (mRS score 0–2) or a poor functional outcome (mRS score 3–6). Stroke severity was assessed by 2 trained neurologists using the NIHSS. If the difference between the two scores exceeded two points, a senior neurologist adjudicated the final NIHSS score.

Inflammatory indices were calculated as follows: NLR = neutrophil count / lymphocyte count; SII = (neutrophil count × platelet count) / lymphocyte count; SIRI = (neutrophil count × monocyte count) / lymphocyte count (17, 18).

### 2.3 Swallowing function assessment

Swallowing function was assessed by a rehabilitation therapist using the Kubota Water Swallow Test (WST) within 24 h of admission. Each patient, seated upright, was asked to drink 30 mL of warm water, and swallowing performance and any cough reflex were observed. The Kubota WST is a validated bedside screening tool that has been widely used in acute stroke populations in East Asia. The WST was graded as follows: Grade 1: Smooth swallowing in one attempt without coughing; Grade 2: Swallowing in more than one attempt without coughing; Grade 3: Swallowing in one attempt with coughing; Grade 4: Swallowing in more than one attempt with coughing; Grade 5: Frequent coughing with incomplete swallowing of the entire volume.

### 2.4 Imaging evaluation

All patients underwent cranial magnetic resonance imaging (MRI) within 48 h of admission using a 3.0 T GE scanner (GE Healthcare, Chicago, IL, USA). MRI sequences included T1-weighted imaging, T2-weighted imaging, diffusion-weighted imaging (DWI), and fluid-attenuated inversion recovery (FLAIR). Additionally, magnetic resonance angiography (MRA) or computed tomography angiography (CTA) was performed to assess the intracranial and basilar artery status.

**Figure 1:**
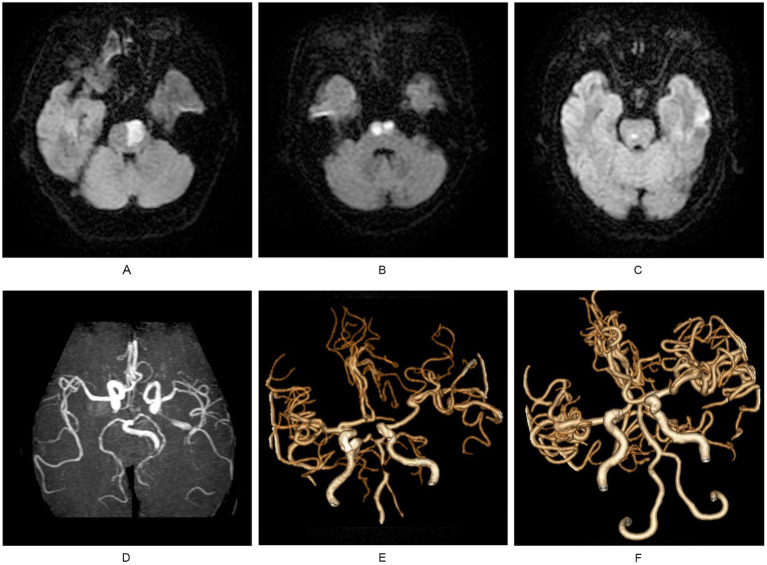
Three etiological subtypes. **(A)** and **(D)**: Basilar artery branch disease (BABD); **(B)** and **(E)**: Large-artery occlusive disease (LAOD); **(C)** and **(F)**: Small-artery disease (SAD).

Based on lesion morphology and the presence of basilar artery stenosis > 50%, AIPI was classified into three etiological subtypes (21): Large-artery occlusive disease (LAOD): basilar artery stenosis > 50%; Basilar artery branch disease (BABD): infarcts extending from the deep pons to the ventral surface with basilar artery stenosis < 50%; Small artery disease (SAD): infarcts confined to the deep pons without ventral surface involvement and with a maximum diameter < 15 mm (Figure 1).

### 2.5 Definition and classification of SAP

SAP was defined as pneumonia occurring within 7 days after stroke onset in non-mechanically ventilated patients and diagnosed according to the modified Centers for Disease Control and Prevention (CDC) criteria for hospital-acquired pneumonia (22, 23). Patients with community-acquired pneumonia or ventilator-associated pneumonia were excluded.

The diagnosis was established by neurologists with specialized training according to the modified CDC criteria and was based on a comprehensive assessment of the following findings: (1) clinical manifestations suggestive of lower respiratory tract infection, such as fever, cough, increased sputum production, or dyspnea; (2) radiological evidence of new or progressive pulmonary infiltrates consistent with infection; (3) laboratory or microbiological findings supporting bacterial infection. Patients were classified into the SAP group or non-SAP group according to whether they met the above diagnostic criteria. Empirical antibiotic treatment was administered after SAP was clinically diagnosed according to routine clinical practice. Infectious disease consultation was arranged at the discretion of the attending neurologist based on individual patient conditions.

### 2.6 Statistical analysis

All statistical analyses were performed using IBM SPSS Statistics version 25.0 (IBM Corp., Armonk, NY, USA). Categorical variables were expressed as frequencies and percentages and compared using the chi-square test or Fisher’s exact test, as appropriate. Continuous variables were tested for normality using the Kolmogorov–Smirnov test. Normally distributed variables were presented as the mean ± standard deviation (SD) and compared using independent-samples t-tests, whereas non-normally distributed variables were expressed as the median (interquartile range, IQR) and analyzed using the Mann–Whitney U test.

Variables with a *p*-value < 0.05 in univariate analyses were entered into a multivariate logistic regression model to identify independent predictors of SAP, with SAP status as the dependent variable. Odds ratios (ORs) and 95% confidence intervals (CIs) were calculated.

Receiver operating characteristic (ROC) curve analysis was performed to evaluate the predictive performance of the WST score, serum albumin level, and SII for SAP. The AUC, sensitivity, specificity, and optimal cutoff values were determined. All tests were two-tailed, and a *p*-value < 0.05 was considered statistically significant.

## 3 Results

### 3.1 Baseline characteristics of the study population

A total of 179 patients with AIPI were included in this study, comprising 117 males (65.36%) and 62 females (34.64%), with a mean age of 69.23 ± 10.67 years. Among them, 19 patients (10.61%) developed SAP, whereas 160 patients (89.39%) did not. When compared with the non-SAP group, patients in the SAP group were significantly older and had higher WST scores, baseline NIHSS scores, fibrinogen levels, CRP levels, NLR, SII, and SIRI (all *p* < 0.05). In contrast, serum albumin levels were significantly lower in the SAP group (*p* < 0.001). No significant differences were observed in etiological subtypes or other baseline demographic and laboratory parameters between the two groups (Table 1). Among patients with SAP, 8 of 19 patients (42.11%) received infectious disease consultation, with a median time of 2.5 days (IQR: 1.0–3.0 days) from SAP diagnosis.

**Table 1:** Comparisons of clinical characteristics between SAP and non-SAP groups.

Characteristics	SAP (*n* = 19)	Non-SAP (*n* = 160)	*P* value
Male, *n* (%)	12 (63.16%)	105 (65.63%)	0.831
Age (years, mean ± SD)	74.53 ± 7.58	68.61 ± 10.82	0.005^*^
Water test score, median [IQR]	3 [1]	1 [1]	<0.001^*^
NIHSS, median [IQR]	4 [5]	2 [3]	<0.001^*^
Vascular risk factors and comorbidities			
Hypertension, *n* (%)	16 (84.21%)	140 (87.50%)	0.716
Diabetes mellitus, *n* (%)	8 (42.11%)	71 (44.38%)	0.851
Atrial fibrillation, *n* (%)	0 (0%)	6 (3.75%)	1.000
Coronary heart disease, *n* (%)	2 (10.53%)	17 (10.63%)	0.674
Cerebral Infarction/TIA, *n* (%)	1 (5.26%)	22 (13.75%)	0.475
Smoking, *n* (%)	7 (36.84%)	54 (32.50%)	0.788
Alcohol, *n* (%)	1 (5.26%)	20 (12.50%)	0.704
Laboratory parameters			
Fibrinogen (mg/mL, mean ± SD)	337.26 ± 84.80	293.06 ± 52.58	0.038^*^
Blood glucose [mmol/L, median (IQR)]	5.27 [3.70]	5.67 [2.78]	0.293
Glycated hemoglobin [%, median (IQR)]	5.80 [3.50]	6.10 [1.90]	0.251
LDL-C [mmol/L, median (IQR)]	2.49 [0.81]	2.49 [0.99]	0.449
Homocysteine [μmol/L, median (IQR)]	14 [5]	12 [5.40]	0.351
Uric acid [μmol/L, median (IQR)]	313 [50]	321 [136.50]	0.822
Albumin (g/L, mean ± SD)	33.17 ± 3.89	38.31 ± 4.49	<0.001^*^
Etiological subtypes			
LAOD, *n* (%)	1 (5.26%)	7 (4.38%)	–
BABD, *n* (%)	13 (68.42%)	78 (48.75%)	0.160
SAD, *n* (%)	5 (26.32%)	75 (46.87%)	–
Inflammatory markers			
CRP, median [IQR]	15.7 [47.30]	2.40 [3.48]	<0.001^*^
NLR, median [IQR]	6.67 [5.97]	2.31 [1.55]	<0.001^*^
SII, median [IQR]	1173.90 [1632.32]	370.67 [302.97]	<0.001^*^
SIRI, median [IQR]	3.40 [5.86]	0.94 [0.80]	<0.001^*^

BABD, branch atheromatous disease; CRP, C-reactive protein; IQR, interquartile range; LAOD, large artery occlusive disease; LDL-C, low-density lipoprotein cholesterol; NIHSS, National Institutes of Health Stroke Scale; NLR, neutrophil-to-lymphocyte ratio; SAD, small artery disease; SAP, stroke-associated pneumonia; SII, systemic immune-inflammation index; SIRI, systemic inflammation response index; TIA, transient ischemic attack. Data are presented as mean ± standard deviation, median [interquartile range], or n (%). *P < 0.05 was considered statistically significant.

### 3.2 Multivariate logistic regression analysis

Multivariate logistic regression analysis identified WST score [odds ratio (OR) = 2.622, 95% confidence interval (CI): 1.378–4.991, *p* = 0.003], serum albumin level (OR = 0.831, 95% CI: 0.735–0.940, *p* = 0.003), and SII (OR = 1.004, 95% CI: 1.002–1.006, *p* < 0.001) as independent predictors of SAP in patients with AIPI. A higher WST score and elevated SII were associated with an increased risk of SAP, whereas a higher serum albumin level was independently protective (Table 2).

**Table 2:** Multivariate logistic regression analysis of SAP in AIPI patients.

Variable	OR	95%CI	*P* Value
Water test score	2.622	1.378–4.991	0.003^*^
Albumin	0.831	0.735–0.940	0.003^*^
SII	1.004	1.002–1.006	<0.001^*^

SAP, stroke-associated pneumonia; AIPI, acute isolated pontine infarction; SII, systemic immune-inflammation index. *P < 0.05 was considered statistically significant.

### 3.3 Predictive performance of the combined model

**Figure 2:**
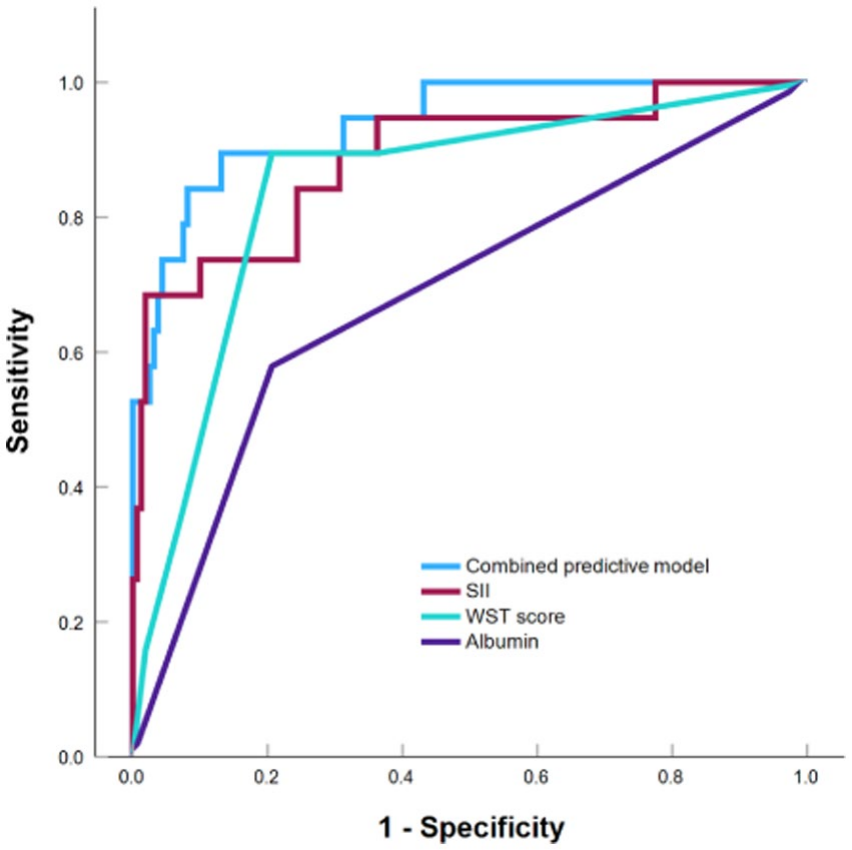
ROC curve for predicting the occurrence of SAP in AIPI patients.

ROC curve analysis demonstrated that the AUC for WST score in predicting SAP was 0.843 (95% CI: 0.747–0.939); for serum albumin, 0.828 (95% CI: 0.752–0.905); and for SII, 0.888 (95% CI: 0.798–0.977). The combined model integrating WST score, serum albumin, and SII achieved an AUC of 0.938 (95% CI: 0.885–0.992), with a sensitivity of 89.5% and specificity of 86.9%, which was superior to any single predictor (Figure 2).

### 3.4 Subgroup analysis according to baseline serum albumin level

An additional subgroup analysis was performed according to baseline serum albumin levels using a cutoff value of 35 g/L. Patients with low albumin levels (< 35 g/L, *n* = 44) had a significantly higher incidence of SAP than those with normal albumin levels (≥ 35 g/L, *n* = 135) (25.0% vs. 5.93%, *p* < 0.001). Regarding 3-month functional outcome, the low-albumin group showed a higher rate of poor functional outcome than the normal-albumin group, although the difference did not reach statistical significance (31.8% vs. 19.3%, *p* = 0.082).

### 3.5 Independent association between SAP and short-term poor prognosis

Patients with AIPI were stratified into favorable and poor prognosis groups according to mRS scores. Multivariate logistic regression analysis demonstrated that SAP was an independent risk factor for short-term poor functional outcome in patients with AIPI (OR = 8.082, 95% CI: 2.922–22.355, *p* < 0.001), indicating that SAP confers a significantly elevated risk of short-term poor prognosis in this patient cohort.

## 4 Discussion

This study found that the incidence of SAP in patients with AIPI was 10.61%. Our results demonstrated that SAP was an independent risk factor for short-term poor functional outcome in this population, which is consistent with the findings of previous studies (2, 4). This is the first study to demonstrate that the WST score, serum albumin level, and SII were independently associated with SAP occurrence. The combination of these indicators significantly improved predictive accuracy, providing new evidence for early risk stratification and preventive management in this high-risk population.

Dysphagia is a well-established risk factor for SAP. Consistent with previous studies (1, 2, 5, 9, 17, 24), our findings confirmed that higher WST scores were significantly associated with SAP development. The reported incidence of dysphagia in pontine infarction ranges from 24 to 43% (25, 26). Although the pons is not the primary swallowing center, its role as an integrative sensory relay within the brainstem means that pontine infarcts can impair pharyngolaryngeal sensation and weaken the cough reflex, thereby increasing the risk of aspiration. In addition, impaired swallowing function may contribute to inadequate nutritional intake and reduced airway protection, further predisposing patients to pulmonary infection (27). Recent studies have emphasized the importance of early screening and structured management of swallowing dysfunction for SAP prevention (28).

The “brain–lung axis” hypothesis proposes a bidirectional neuroendocrine and immune regulatory connection between the central nervous system and the lungs (29). After stroke, inflammatory mediators released from damaged brain tissue can cross a disrupted blood–brain barrier, triggering systemic inflammatory activation and peripheral immunosuppression (30, 31). This imbalance disturbs brain–lung immune homeostasis and increases susceptibility to pulmonary infection (32). Elevated SII reflects neutrophil activation, lymphocyte depletion, and platelet involvement in systemic inflammation. Within the context of brain–lung axis dysregulation, SII serves as a quantitative biomarker of systemic immune–inflammatory status and has been shown to be independently associated with SAP (33, 34).

Our results also demonstrated that lower serum albumin levels were independently associated with a higher risk of SAP, consistent with previous studies (19, 35, 36). The potential protective mechanisms of albumin include antioxidant activity via free thiol groups (37), modulation of pro-inflammatory cytokine release (38), and regulation of neutrophil NADPH oxidase activity, thereby enhancing innate immune efficiency (39). Beyond its biological effects, serum albumin is also a well-recognized marker of nutritional status and systemic resilience in acute illness. Hypoalbuminemia often reflects poor nutritional status, which has been shown to have a dose–response relationship with SAP risk (40).

A recent meta-analysis confirmed that the neutrophil-to-lymphocyte ratio (NLR) is a useful, inexpensive biomarker for predicting post-stroke pneumonia (41). In our univariate analysis, NLR was significantly higher in SAP patients, consistent with this finding. However, in multivariate regression, NLR was not independently associated with SAP after adjusting for WST score, albumin, and SII—likely due to the relatively small sample size, the specific AIPI population, and the stronger predictive performance of SII in our model. Nevertheless, an elevated NLR may still serve as a low-cost bedside marker to identify patients at increased risk of post-stroke infection and to prompt closer clinical monitoring.

The novelty of this study lies in the first integration of WST score, serum albumin level, and SII for predicting SAP risk in patients with AIPI. Each of these indicators alone showed good predictive performance, and the combined model further enhanced diagnostic accuracy, achieving an AUC of 0.938. This was superior to most conventional clinical prediction models, such as the A2DS2, ISAN, and AIS-APS scores (AUC: 0.7–0.85) (13, 14, 42). Compared with existing models that primarily rely on clinical variables, our model integrates functional, nutritional, and inflammatory dimensions, thereby providing a more comprehensive assessment of SAP risk (AUC: 0.855–0.924) (43). Furthermore, all three predictors are readily available, inexpensive, and suitable for use across different healthcare settings, making this approach practical for widespread clinical application.

Several limitations should be acknowledged. First, swallowing function was assessed using the Kubota WST rather than instrumental assessments such as video fluoroscopic swallowing study (VFSS) or fiberoptic endoscopic evaluation of swallowing (FEES). Second, SII levels may be influenced by concurrent inflammatory or stress-related conditions that were not fully controlled in this retrospective design. Although previous studies have assessed post-stroke pneumonia severity using tools such as the CURB-65 score, pneumonia severity scores were not systematically available in the present retrospective cohort (44). Third, sputum culture, pathogen detection and bacterial typing were not routinely conducted in all patients with stroke-associated pneumonia during hospitalization; thus, we failed to systematically analyze differences in bacterial pathogen types and their potential impact on functional outcomes.

In conclusion, this study demonstrated that the WST score, serum albumin level, and SII are independent predictors of SAP in patients with AIPI. The combined use of these readily available indicators may facilitate early identification of high-risk patients and support timely preventive interventions in clinical practice.

## 5 Conclusion

This study identified the WST score, serum albumin level, and systemic immune-inflammation index (SII) as independent predictors of SAP in patients with AIPI. Based on these findings, we propose that clinical practice consider adopting a combined screening model integrating these three parameters. This approach would facilitate the early identification of high-risk patients, enabling timely preventive interventions and potentially reducing the incidence of adverse outcomes.

## Data Availability

The raw data supporting the conclusions of this article will be made available by the authors, without undue reservation.
